# Mutation Spectrum of Common Deafness-Causing Genes in Patients with Non-Syndromic Deafness in the Xiamen Area, China

**DOI:** 10.1371/journal.pone.0135088

**Published:** 2015-08-07

**Authors:** Yi Jiang, Shasha Huang, Tao Deng, Lihua Wu, Juan Chen, Dongyang Kang, Xiufeng Xu, Ruiyu Li, Dongyi Han, Pu Dai

**Affiliations:** 1 Department of Otolaryngology, Head and Neck Surgery, PLA General Hospital, Beijing, P. R. China; 2 Fujian Medical University ShengLi clinical college, Fujian Provincial Hospital, Fuzhou, P. R. China; 3 Beijing Capital Bio Independent Clinical Laboratory, Beijing, P. R. China; Kunming Institute of Zoology, Chinese Academy of Sciences, CHINA

## Abstract

In China, approximately 30,000 babies are born with hearing impairment each year. However, the molecular factors causing congenital hearing impairment in the Xiamen area of Fujian province have not been evaluated. To provide accurate genetic testing and counseling in the Xiamen area, we investigated the molecular etiology of non-syndromic deafness in a deaf population from Xiamen. Unrelated students with hearing impairment (*n* = 155) who attended Xiamen Special Education School in Fujian Province were recruited for this study. Three common deafness-related genes, *GJB2*, *SLC26A4*, and *mtDNA12SrRNA*, were analyzed using all-exon sequencing. *GJB2* mutations were detected in 27.1% (42/155) of the entire cohort. The non-syndromic hearing loss (NSHL) hotspot mutations c.109G>A (p.V37I) and c.235delC were found in this population, whereas the Caucasian hotspot mutation c.35delG was not. The allelic frequency of the c.109G>A mutation was 9.03% (28/310), slightly higher than that of c.235delC (8.39%, 26/310), which is the most common *GJB2* mutation in most areas of China. The allelic frequency of the c.109G>A mutation was significantly higher in this Xiamen’s deaf population than that in previously reported cohorts (P = 0.00). The *SLC26A4* mutations were found in 16.77% (26/155) of this cohort. The most common pathogenic allele was c.IVS7-2A>G (6.13%, 19/310), and the second most common was the c.1079C>T (p.A360V) mutation (1.94%, 6/310) which has rarely been reported as a hotspot mutation in other studies. The mutation rate of *mtDNA12SrRNA* in this group was 3.87% (6/155), all being the m.A1555G mutation. These findings show the specificity of the common deaf gene-mutation spectrum in this area. According to this study, there were specific hotspot mutations in Xiamen deaf patients. Comprehensive sequencing analysis of the three common deaf genes can help portray the mutation spectrum and develop optimal testing strategies for deaf patients in this area.

## Introduction

Hearing impairment is among the most common congenital disorders; approximately half of the cases are caused by genetic factors. In China, many previous genetic screening studies have shown that *GJB2*, *SLC26A4*, and *mtDNA12SrRNA* are the three most common causes of non-syndromic deafness, and the mutations in these three genes can explain 26.65–35.7% of the pathogenesis of Chinese non-syndromic hearing impairment [1–3]. Mutations in *GJB2* have been reported to be the most common molecular defect in the Chinese deaf population, with an almost 21% mutation detection rate; of these mutations, c.235delC is the most common in the Chinese deaf population [4,5]. The most common mutation of *SLC26A4* seen in the Chinese deaf population is c.IVS7-2A>G; its detection rate can be as high as 12.5% [6–8]. The most common mutation of *mtDNA12SrRNA* is m.A1555G, the frequency of which was reported to be 3.43% in a Chinese deaf population [9].

China is a large country with the highest population in the world. Chinese people from different areas may have different genetic backgrounds due to geographical and language separation [10]. Xiamen is a city in the South Fujian region in southern China, neighboring the Golden Gate Island of Taiwan. Because it is an island, Xiamen communicated with the mainland by boat and it was relatively isolated before the construction of Xiamen Gaoji Seawall in 1955. The most ancient Xiamen resident migrated from the Chinese Central Plains region due to the wars during the Chinese Shang, Tang, and Song Dynasties, and maintained their language (now called Hokkien or Southern Min) and traditional culture. The close-relative marriage rule can be traced back to ancient China, where intermarriages among individuals with the same surname or among clansmen were forbidden or not encouraged. However, the phenomenon of marriage among individuals who have the same language and culture prevailed [11]. Until now, no systematic genetic analysis of deaf patients in Xiamen has been reported previously. To provide accurate genetic testing and counseling in the Xiamen area, we evaluated the molecular etiology of non-syndromic deafness in a deaf population from Xiamen Special Education School. In total, 155 patients with severe-to-profound hearing loss were recruited for this investigation. All exons in the open reading frames (ORF) of the *GJB2*, *SLC26A4*, and *mtDNA12SrRNA* genes were sequenced and analyzed.

## Materials and Methods

### Patients and DNA samples

In total, 155 unrelated patients with hearing impairment (152 students, three teachers) from Xiamen Special Education School in Fujian province were recruited for this study. The patient cohort consisted of 93 males and 62 females, 5–36 years old (13.40 years, on average): in terms of ethnicity, the cohort comprised 153 Han and two She. Hearing tests demonstrated that the level of hearing loss was severe to profound in all cases. There were 25 patients whose families had more than one other family member with a hearing impairment. No family had a consanguineous marriage except that one patient’s parents were cousin.

The protocol for this investigation was performed with the approval of the ethics committees of the Chinese PLA General Hospital and Fujian Provincial Hospital. The notification of deafness gene detection was performed a half month prior. We provided the consent form and individual information questionnaire to patients and guardians who volunteered to participate in this detection study. We obtained informed consent from all participants; in the case of minors, consent was obtained from their parents/guardians on their behalf. All of the subjects or their guardians signed the informed consent forms prior to blood sampling. Questionnaires included basic information, including name, age, address, family history, health records of the mother during pregnancy, and a clinical history of the patient, such as infections, possible head or brain injury, and the use of aminoglycoside antibiotics. All subjects underwent hearing tests and medical examinations. DNA was extracted from peripheral blood leukocytes using a commercially available DNA extraction kit (Watson Biotechnologies Inc., Shanghai, China).

### Variant analysis

The coding exons plus approximately 50–100 bp of the flanking intron regions of *GJB2*, *SLC26A4*, and *mtDNA12SrRNA* were amplified by polymerase chain reaction (PCR), and Sanger sequencing was used to determine the sequences in all patients. According to the manufacturer’s manual, the results were analyzed using an ABI 3100 DNA sequencing machine (ABI, Foster City, CA, USA) and the ABI 3100 Analysis Software (ver. 3.7 NT). Patients with monoallelic *GJB2* coding region mutations were further tested for the *GJB2* c.IVS1+1G>A mutation or defects in *GJB2* exon 1 and its basal promoter [5,6]. Data analysis was performed using SPSS20.0 software.

## Results

### *GJB2*

Fourteen variants were identified in this cohort. Seven of them were pathogenic including three frameshift deletions (c.235delC, c.176del16, c.299delAT), one frameshift insertion (c.512insAACG), and three missense mutations [c.109G>A(p.V37I), c.257C>G(p.T86R), c.187G>T(p.V63L)]. The mutant alleles of *GJB2* accounted for 20.0% (62/310) of the total alleles in the 155 NSHL patients(Table 1). Unlike most areas of China, the most common mutation allele of GJB2 in Xiamen was not c.235delC but c.109G>A. The allele frequency of c.109G>A was 9.03% (28/310), followed by 8.39% (26/310) for c.235delC, 0.65% (2/310) for c.257C>G, 0.65% (2/310) for c.299delAT, 0.65% (2/310) for c.512insAAGG, 0.32% (1/310) for c.176del16, and 0.32% (1/310) for c.187G>A (Table 2). There were seven types of GJB2 polymorphisms: c.11G>A, c.34G>T, c.79G>A, c.341A>G, c.368C>A, c.571T>C, and c.608T>C. All of the variants were reported and discussed in previous studies.

**Table 1:** *GJB2* genotypes of deaf patients from Xiamen special education school.

Allele 1			Allele 2			Number of patients
Nucleotide change	Consequence or amino acid change	Category	Nucleotide change	Consequence or amino acid change	Category	
c.109G>A[12]	p.V37I	Pathogenic	c.109G>A	p.V37I	Pathogenic	4
c.176del16[5]	Frameshift	Pathogenic	c.257C>G[5]	p.T86R	Pathogenic	1
c.235delC[5]	Frameshift	Pathogenic	c.235delC	Frameshift	Pathogenic	9
c.235delC	Frameshift	Pathogenic	c.299delAT[13]	Frameshift	Pathogenic	2
c.235delC	Frameshift	Pathogenic	c.257C>G	p.T86R	Pathogenic	1
c.235delC	Frameshift	Pathogenic	c.512insAACG	Frameshift	Pathogenic	1
c.235delC	Frameshift	Pathogenic	c.109G>A	p.V37I	Pathogenic	1
c.512insAACG[5]	Frameshift	Pathogenic	c.109G>A	p.V37I	Pathogenic	1
c.109G>A	p.V37I	Pathogenic				12
c.109G>A	p.V37I	Pathogenic	c.34G>T[5]	p.G12D	Polymorphism	1
c.109G>A	p.V37I	Pathogenic	c.79G>A,c.341A>G[14]	p.V27I,p.E114G	Polymorphism	4
c.109G>A	p.V37I	Pathogenic	c.571T>C[15]	p.F191L	Polymorphism	1
c.187G>T[13]	p.V63L	pathogenic				1
c.235delC	Frameshift	Pathogenic	c.79G>A,c.341A>G	p.V27I,p.E114G	Polymorphism	1
c.235delC	Frameshift	Pathogenic	c.79G>A	p.V27I	Polymorphism	1
c.235delC	Frameshift	Pathogenic	c.608T>C[14]	p.I203T	Polymorphism	1
c.11G>A[16]	p.G4D	Polymorphism				1
c.79G>A,c.341A>G	p.V27I,p.E114G	Polymorphism				26
c.79G>A,c.341A>G	p.V27I,p.E114G	Polymorphism	c.79G>A,c.341A>G	p.V27I,p.E114G	Polymorphism	10
c.79G>A,c.341A>G	p.V27I,p.E114G	Polymorphism	c.608T>C	p.I203T	Polymorphism	5
c.79G>A	p.V27I	Polymorphism	c.608T>C	p.I203T	Polymorphism	3
c.79G>A	p.V27I	Polymorphism	c.368C>A	p.T123N	Polymorphism	1
c.79G>A	p.V27I	Polymorphism				8
c.341A>G	p.E114G	Polymorphism				1
c.571T>C	p.F191L	Polymorphism				1
c.608T>C	p.I203T	Polymorphism				9

**Table 2:** Allele Frequencies of GJB2 mutation in 155 deaf patients from Xiamen special education school.

	Mutations in the genes	Consequence or amino acid change	Number of alleles	Alleles Frequency (%)	Reference
*GJB2*	c.109G>A	p.V37I	28	9.03	16
	c.235delC	Frameshift	26	8.39	5
	c.257C>G	p.T86R	2	0.65	5
	c.299delAT	Frameshift	2	0.64	35
	c.512insAAGG	Frameshift	2	0.65	5
	c.176del116	Frameshift	1	0.32	5
	c.187G>A	p.V63L	1	0.32	35

There were 20 patients (12.9%) confirmed to have GJB2 deafness-causing mutations: 13 homozygotes (nine with the c.235delC allele and four with the c.109G>A allele) and seven compound heterozygotes. Twenty-two patients (14.2%) carried only one heterozygous pathogenic mutation: one with c.187G>T, three with c.235delC and eighteen with c.109G>A. Thus, the detection rate of GJB2 mutations in the patients was 27.1% (42/155) (Table 1).

Among the 24 patients carrying at least one c.109G>A allele, there were four homozygotes, one compound heterozygote with c.235delC, one compound heterozygote with 512insAACG, and 18 single heterozygotes. The second most common mutation of *GJB2* in this group was c.235delC, with a mutation allele frequency of 8.39% (26/310): nine homozygotes, five compound heterozygotes, and three heterozygotes.

### *SLC26A4*

Twenty-two variants were identified in this cohort, including three novel variants (c.1167G>A, c.1738_1739delAA, and c.1764_1765insAGGAAAATA). Among the four splice site variants in this cohort, c.IVS7-2A>G and c.IVS16-6G>A are pathogenic because they affect canonical splice donor and acceptor nucleotide positions that had been previously identified; c.IVS11+47T>C was previously identified as a polymorphism, and c.IVS7-18T>C was considered a polymorphism in our laboratory, which will be analyzed further in the Discussion section. The three frameshift insertions [c.916_917insG (FS306, P329*), c.1692_1693insA (FS565, P573*), and c.1764_1765insAGGAAAATA (FS558)], two truncated mutations [c.2086C>T (p.Q696*) and c.1336C>T (p.Q446*)], and one frameshift deletion [c.1738_1739delAA (FS580, P606*)] were considered pathogenic due to a dramatic prematurely altered or truncated protein. Combining the prediction of SIFT and Polyphen-2 with the results of previous studies, the following 12 missense variants were analyzed in our cohort: c.1079C>T (p.A360V), c.2168A>G (p.H723R), c.754T>C (p.S252P), c.1229C>T (p.T410M), c.1472T>C (p.I491T), c.1595G>T (p.S532I), and c.2007C>G (p.D669E) were considered pathogenic mutations, and c.1790T>C (p.L597S) and c.147C>G (p.S49R) were deemed polymorphisms. c.2283A>G (p.T761T) and c.1167G>A (p.G389G, this study) were considered polymorphisms without amino acid sequence alteration after the nucleotide changed. c.2009T>C (p.V670A) still had unknown significance according to our data and prediction, which will be discussed later (Table 3).

**Table 3:** *SLC26A4* genotypes of deaf patients from Xiamen special education school.

Allele 1			Allele 2			Number of patients
Nucleotide change	Consequence or amino acid change	Category	Nucleotide change	Consequence or amino acid change	Category	
c.754T>C[17]	p.S252P	Pathogenic	c.1738_1739delAA	FS580,P606*	Pathogenic	1
c.916_917insG[18]	FS306,P329*	Pathogenic	c.2168A>G[19]	p.H723R	Pathogenic	1
c.IVS7-2A>G[7]	aberrant splicing	Pathogenic	c.IVS7-2A>G	aberrant splicing	Pathogenic	5
c.IVS7-2A>G	aberrant splicing	Pathogenic	c.1079C>T[20]	p.A360V	Pathogenic	3
c.IVS7-2A>G	aberrant splicing	Pathogenic	c.2086C>T[21]	p.Q696*	Pathogenic	2
c.IVS7-2A>G	aberrant splicing	Pathogenic	c.1336C>T[7]	p.Q446*	Pathogenic	1
c.IVS7-2A>G	aberrant splicing	Pathogenic	c.2007C>G[22]	p.D669E	Pathogenic	1
c.IVS7-2A>G	aberrant splicing	Pathogenic	c.2168A>G	p.H723R	Pathogenic	1
c.1079C>T	p.A360V	Pathogenic	c.1079C>T	p.A360V	Pathogenic	1
c.1229C>T[17]	p.T410M	Pathogenic	c.2168A>G	p.H723R	Pathogenic	1
c.1692_1693insA[6]	FS565,P573*	Pathogenic	c.2168A>G	p.H723R	Pathogenic	1
c.147C>G[23]	p.S49R	pathogenic				1
c.IVS7-2A>G	aberrant splicing	Pathogenic				1
c.1472T>C[7]	p.I491T	Pathogenic				1
c.1595G>T[6]	p.S532I	Pathogenic				1
c.IVS16-6G>A [24]	aberrant splicing	Pathogenic				2
c.IVS16-6G>A	aberrant splicing	Pathogenic	c.IVS11+47T>C[25]	splice site variant	Polymorphism	1
c.1764_1765insAGGAAAATA	Frameshift	Pathogenic				1
c.2009T>C[23]	p.V670A	Unknown				2
c.IVS7-18T>G[26]	splice site variant	Polymorphism				3
c.IVS7-18T>G	splice site variant	Polymorphism	c.IVS11+47T>C	splice site variant	Polymorphism	1
c.1167G>A	p.G389G	Silent variants				1
c.IVS11+47T>C	splice site variant	Polymorphism	c.IVS11+47T>C	splice site variant	Polymorphism	4
c.IVS11+47T>C	splice site variant	Polymorphism				23
c.1790T>C[27]	p.L597S	Polymorphism				1
c.2283A>G[6]	p.T761T	Silent variants				4

Note:

*: stop codon

There were 18 (11.61%) patients confirmed to be carrying two *SLC26A4* pathogenic mutations: six homozygotes (5 c. IVS 7-2A>G and 1 c.1079C>T), twelve compound heterozygotes, and eight (5.16%) patients with one *SLC26A4* pathogenic mutation. Thus, the detection rate of *SLC26A4* mutations was 16.77% (26/155) in this patient cohort.

The most common mutation of *SLC26A4* was c.IVS7-2A>G, with a 6.13% (19/310) allele frequency. Except for five homozygotes and one heterozygote of it, there were eight patients carrying c.IVS7-2A>G heterozygous mutations combined with the other mutations included three c.1079C>T, two c.2086C>T, one c.2168A>G, one c.1336C>T, and one c.2007C>G.

The mutation allele frequency was 1.61% (5/310) for c.1079C>T, 1.29% (4/310) for c.2168A>G, 0.97% (3/310) for c. ivs16-6G>A, 0.65% (2/310) for c.2086C>T and 0.32% (1/310) for each of the others (Table 4).

**Table 4:** Allele Frequencies of *SLC26A4* mutation in 155 deaf patients from Xiamen special education school.

	Mutations in the genes	Consequence or amino acid change	Number of alleles	Alleles Frequency (%)	Reference
*SLC26A4*	c.IVS7-2A>G	aberrant splicing	19	6.13	7
	c.1079C>T	p.A360V	5	1.61	23
	c.2168A>G	p.H723R	4	1.29	21
	c.IVS16-6G>A	aberrant splicing	3	0.97	43
	c.2086C>T	p.Q696*	2	0.65	41
	c.754T>C	p.S252P	1	0.32	39
	c.916_917insG	FS306,P329*	1	0.32	40
	c.1229C>T	p.T410M	1	0.32	39
	c.1336C>T	p.Q446*	1	0.32	7
	c.1472T>C	p.I491T	1	0.32	7
	c.1595G>T	p.S532I	1	0.32	6
	c.1692_1693insA	FS565,P573*	1	0.32	6
	c.2007C>G	p.D669E	1	0.32	42
	c.1738_1739delAA	FS580,606*	1	0.32	This study
	c.1764_1765insAGGAAAATA	Frameshift	1	0.32	This study

Note:

*: stop codon

### Mutation analysis

#### c.IVS7-18T>C

This variant has been proposed to exert a likely pathogenic effect according to the reported data (http://deafnessvariationdatabase.org/). According to the Fruitfly analysis tool (http://www.fruitfly.org/seq_tools/splice.html), a change in the splice donor sequence from T to C in intron 7 (c. IVS7-18T>C) of SLC26A4 is predicted to make no difference in splice site recognition by the splicing factor from 0.93 to 0.92 (when the score is reduced to less than 0.4, the mutation may affect the function of the original). We checked the previous records of our laboratory and found that four of 261 control subjects (1.53%) carried the single heterozygous c.IVS7-18T>C allele. Based on prediction analysis and our laboratory data, we concluded that this variation was a polymorphism.

#### c.2009T>C (p.V670A)

**Fig 1:**
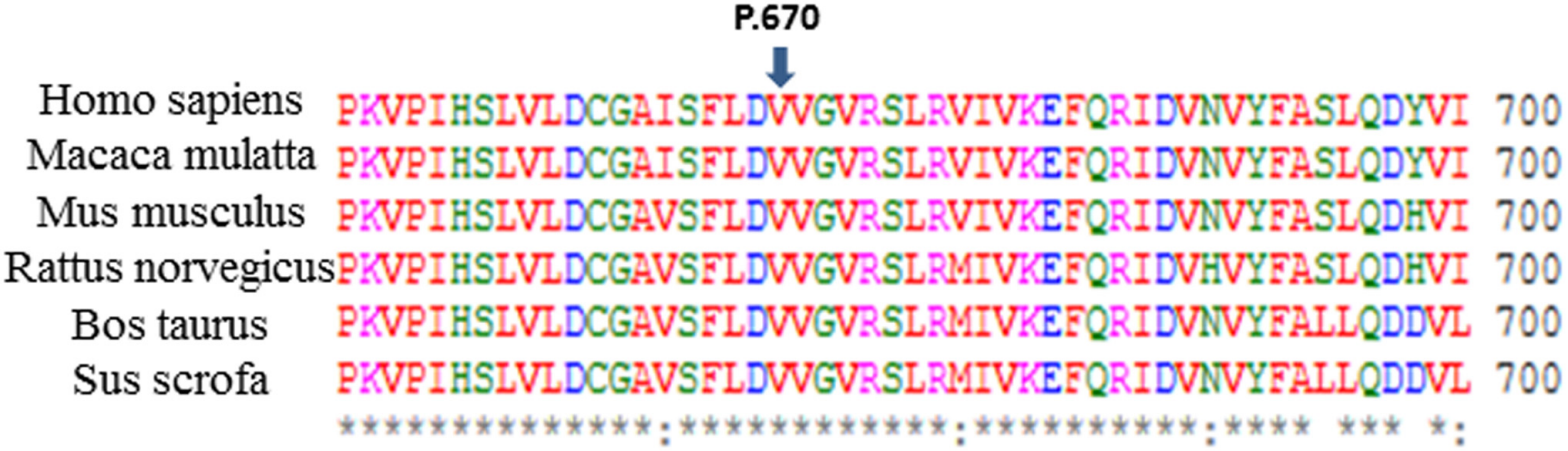
Protein alignment showing conservation of residues GJB2 V670 across six species. An alignment of the SLC26A4 amino acid sequence of six species suggested the evolutionary conservation of c.2009T>C (p.V670A).

This variant was not found in 1,668 EVAS patients from our laboratory before this study, and the mutation frequency of c.2009T>C in this Xiamen’s cohort was 1.29% (2/155). We predicted the pathogenicity of this mutation by SIFT and Polyphen-2, and the results suggested “tolerant,” with a SIFT score of 0.05 and “possibly damaging” with a Polyphen-2 score of 0.873 by each. We made an alignment of the SLC26A4 amino acid sequence of six species, namely *Homo sapiens* (043511), *Macaca mulatta* (XP_001094049.1), *Mus musculus* (NP_035997.1), *Rattus norvegicus* (NP_062087.1), *Bos taurus* (XP_608706.3), and *Sus scrofa* (XP_003357559.1) (Fig 1), and the results suggested the evolutionary conservation of c.2009T>C. However, only two of 155 patients (1.29%) were found to carry the heterozygote of this variant in the present study; thus, a conclusion still cannot be reached.

### *mtDNA12SrRNA*

Six patients (3.87%) carried a *mtDNA12SrRNA* mutation, all of which were the m.1555A>G homoplasmic mutation. Four patients had a clear history of aminoglycoside antibiotic use. Among them, one used gentamicin alone, one used streptomycin alone, one combined gentamicin and streptomycin, and one combined streptomycin and neomycin.

## Discussion

### *GJB2*

In this study, *GJB2* mutations were detected in 27.1% (42/155) of all patients, with 12.9% (20/155) having two pathogenic mutations and 14.2% (22/155) having only one mutant allele. c.109G>A (p.V37I) and c.235delC were the hotspot mutations in the non-syndromic hearing loss (NSHL) patients in the Xiamen area, whereas the Caucasian hotspot mutation c.35delG [28,29] was not detected. Unlike other areas of China, c.235delC (8.39%) was not the most common GJB2 mutation, but rather c.109G>A, the allelic mutation frequency of which was 9.03% (28/310), indicating some features of GJB2 mutations in this area.

In East Asians, the c.109G>A (p.V37I) mutation is common: the frequency of this allele in the deaf population had been reported to be 4.3% in Thailand [30], 0.6% in Korea [31], 1.0% in Japan [32] and 4.2%(175/4162) in China [5]. Li reported that the frequency of c.109G>A mutation in newborns was 6.2% in Shanghai, China [12]. Given that some individuals with normal hearing also carry the homozygous c.109G>A mutation, there has been much controversy concerning its significance [33,34]. Recent research has shown that the hearing phenotypes of homozygous c.109G>A patients could be diverse in Chinese Han, and such patients may even show severe to profound hearing loss [35]. In our group, the four patients with the homozygous c.109G>A mutation had severe-to-profound hearing loss, the severity of which was similar to that of others in this cohort. In our study, the allele frequency of the c.109G>A mutation was 9.03% (28/310). Compared with the former data (4.2%, 175/4126) from the large Chinese deaf population evaluated by Dai [5], the frequency of the c.109G>A mutation in Xiamen was remarkably higher than that obtained in Dai’s results (P = 0.00). Because the c.109G>A mutation has been found in many countries in Southeast Asia, Oceania, East Asia, North Africa, Eastern Europe, and the Middle East, the complexity of its origin and migration should be considered. Furthermore, it will be worthwhile to study the founder effect and migration route in China based upon the large-sized samples from representative areas of China that could explain the significant differences in the frequency of c.109G>A among the studies of different Chinese deaf populations [36].

In East and Southeast Asia, c.235delC has been reported as the most common mutation acting as a frameshift mutation causing premature protein termination in hearing impaired patients, while lower frequencies were reported in Europe and Oceania [19–31]. In an earlier nationwide study by our group, Dai analyzed the GJB2 mutation of 2063 unrelated NSHL students from 23 different regions of China, the c.235delC mutation allele frequency was 12.34% (509/4,126)[5]. Compared with the 8.39% (26/310) c.235delC mutation allele frequency in the Xiamen area, the difference in the c.235delC mutation allele frequency was not significant (P = 0.064), reflecting a certain consistency of the c.235delC frequency between the Xiamen and entire nation. The shift in the c.235delC mutation from the most common to the second most common was a feature of the Xiamen deaf population that might indicate that, among the Xiamen aboriginals, the carrying rate of c.235delC was likely lower than the carrying rate of c.109G>A in Xiamen.

The c.512insAACG mutation was first reported by Dai; it is a frameshift mutation with a mutation allele frequency of 0.58% (12/2,063) in a Chinese population [5]. In our group, two patients were c.512insAACG compound heterozygotes with c.235delC and c.109G>A, respectively, resulting in a 0.65% (2/310) mutation allele frequency.

### *SLC26A4*

Pendrin, a 780-amino acid protein, functions as a transmembrane anion exchanger and is encoded by *SLC26A4*. Over 100 mutations in the *SLC26A4 (PDS)* gene are involved in both Pendred syndrome (PS) and NSHL (DFNB4), showing specific differences among racial backgrounds. The mutation hot spots are p.T416P and c.IVS8+1G>A in Northern Europe [37], versus p.H723R and c. IVS7-2A>G in South Korea [19] and p.H723R in Japan [38]. It was reported by Dai that the most common Chinese *SLC26A4* mutation is c.IVS7-2A>G, the mutation allele frequency reported in that study was 8.65% (566/6542)[8]. In our study, whole exons of *SLC26A4* were sequenced, and the most common mutation in our Xiamen patient cohort was also c.IVS7-2A>G, the mutation allele frequency was 6.13% (19/310). The frequencies of c.IVS7-2A>G found in Xiamen’s deaf population and in the large Chinese deaf population evaluated by Dai [8] are not significantly different (P = 0.12).

c.1079C>T (p.A360V), the second most common mutation of *SLC26A4* in the Xiamen patient cohort, was first reported in Taiwan by Lai et al. in 2007, with two compound heterozygous missense mutations involving a residue in a highly conserved domain of the eighth transmembrane region [20]. One of the patients in Taiwan with a compound heterozygous mutation of c.1079C>T and c.IVS7-2A>G was positive for both the perchlorate discharge test (PDT) and anti-thyroid peroxidase antibodies (anti-TPO Abs), suggestive of Pendred’s syndrome. This mutation has rarely been reported in other studies. In our group, there was one homozygous mutation and three compound heterozygous mutations of c.1079C>T and c.IVS7-2A>G, but none of the patients had thyroid dysfunction. The four patients were aged 17, 11, 15, and 18 years, respectively, and the onset ages of deafness were 2, 3, 2, and 3.4 years, respectively. Compared with the previous studies by Dai [7]and Guo [1] in China, c.1079C>T was not found in 647(135+514) patients and 167(50+117) normal control subjects. However, one study by Yuan [6] in China found only one heterozygote in their large cohort of 2,352 patients and none in the normal control group. The phenomenon of c.1079C>T mostly being found in Xiamen and Taiwan, but very rarely occurring in other areas, suggested the nature of the distribution of this mutation. Previous investigations of multiple regions of China did not include the Southern Fujian people, whose dialect is Hokkien (Southern Min). Hokkien is one of the eight main Chinese dialects that are popularly spoken in Taiwan and Xiamen. Historically, a proportion of Taiwan's population migrated from southern China (mostly from Xiamen and Quanzhou). This may partly explain why c.1079C>T has mostly been found in Xiamen and Taiwan; however, more samples from such areas will be needed to analyze the origin and migration of c.1079C>T.

c.2168A>G (p.H723R) is a common mutation in Japanese and Korean populations, with mutation allele frequencies of 4.1% and 10.34%[39–41], respectively. The c.2168A>G allele frequency was reported to be 1.51%(71/4704)in China [6]. In our report, the mutation allele frequency was 1.29% in the Xiamen area. The allele frequency of c.2168A>G in this study is consistent with the allele frequency of this mutation in Yuan’s study [6]. Our study suggested differences in the c.2168A>G frequency among countries and areas. Haplotype analyses in previous studies confirmed that c.2168A>G was a founder mutation in Japan, Korea and China, but the geographical environment and migration route may suggest explanations for the significant gradient distribution of c.2168A>G in these three East Asian countries [36].

### *mtDNA12SrRNA*

Associated with aminoglycoside antibiotic-induced deafness [42], the *mtDNA12SrRNA* mutation shows variation by racial and geographic origins in populations with non-syndromic hearing loss, with a frequency of 0.6-2.5% in Caucasians [43–47], 2.4% in Danes [44], 1.8% in Turks [45], 0.7% in Germans, 1.8% in Hungarians, and 2.4% in Poles [43]. It also showed frequencies of 3% and 5.3% in Japanese and Indonesian deaf populations, respectively [44,45]. The m.1555A>G mutation is a common mutation of the mitochondrial 12SrRNA gene, shown to be the third most common mutation in China with a frequency of 3.43%[9]. In this study, the 3.87% (6/155) frequency of m.A1555G was close to that observed by Dai (3.43%).

## Conclusions

In this study, the three most common deafness-associated genes were investigated by exon sequencing in a cohort of deaf patients recruited from Xiamen Special Education School. According to exon sequencing of *GJB2*, *SLC26A4*, and *mtDNA12SrRNA*, almost half of the deaf cases appeared to have a genetic etiology. Our data revealed special hotspots and spectra of mutations in the Xiamen deaf population, and this information will be helpful in designing the protocol for genetic testing for deafness and achieving an accurate molecular diagnosis in the Xiamen area.

## Supporting Information

S1 Table The silico pathogenicity prediction of missense variations (DOCX)
