# Supplementary material for: Mutation Spectrum of Common Deafness-Causing Genes in Patients with Non-Syndromic Deafness in the Xiamen Area, China
Source: PLoS One. 2015 Aug 7;10(8):e0135088. doi: 10.1371/journal.pone.0135088 (PMC4529078; doi:10.1371/journal.pone.0135088)
Supplement: S1 Table — (DOCX) [file pone.0135088.s001.docx]

Supplementary Table.1 The silico pathogenicity prediction of missense variations

|  | missense variations | SIFT/score | Plyphen-2/score | Deafness variation database | minor allele frequency |
| --- | --- | --- | --- | --- | --- |
| *GJB2* | c.11G>A (p.G4D) | TOLERANT /0.16 | Benign/0.088 | Benign | 0.0002 |
|  | c.34G>T(p.G12D) | TOLERANT /0.10 | probably damaging/1.000 | Benign | Not available |
|  | c.79G>A(p.V27I) | TOLERANT /0.25 | probably damaging/1.000 | Benign | 0.0719 |
|  | c.109G>A(p.V37I) | TOLERANT /1.00 | probably damaging/1.000 | Pathogenic | 0.0154 |
|  | c.187G>T(p.V63L) | DAMAGING/0.04 | probably damaging/1.000 | Pathogenic | Not available |
|  | c.257C>G(p.T86R) | DAMAGING/0.01 | probably damaging/1.000 | Pathogenic | Not available |
|  | c.341A>G(p.E114G) | TOLERANT /0.41 | Benign/0.001 | Benign | 0.0310 |
|  | c.368C>A(p.T123N) | TOLERANT /0.53 | Benign/0.000 | Benign | 0.0018 |
|  | c.571T>C(p.F191L) | DAMAGING/0.01 | probably damaging/1.000 | Unknown significance | Not available |
|  | c.608T>C(p.I203T) | DAMAGING/0.00 | probably damaging/0.906 | Benign | 0.0602 |
| *SLC26A4* | c.147C>G (p.S49R) | TOLERANT /1.00 | Benign/0.000 | Likely benign | Not available |
|  | c.754T>C(p.S252P) | DAMAGING/0.01 | probably damaging/1.000 | Pathogenic | Not available |
|  | c.1079C>T(p.A360V) | DAMAGING/0.00 | probably damaging/1.000 | Pathogenic | Not available |
|  | c.2168A>G(p.H723R) | DAMAGING/0.00 | probably damaging/1.000 | Pathogenic | 0.0004 |
|  | c.1229C>T(p.T410M) | DAMAGING/0.00 | probably damaging/1.000 | Pathogenic | 0.0002 |
|  | c.1472T>C(p.I491T) | DAMAGING/0.01 | probably damaging/0.749 | Pathogenic | Not available |
|  | c.1595G>T(p.S532I) | DAMAGING/0.00 | Benign/0.399 | Pathogenic | Not available |
|  | c.1790T>C( p.L597S) | TOLERANT /0.08 | probably damaging/0.999 | Benign | 0.0086 |
|  | c.2007C>G(p.D669E) | DAMAGING/0.00 | probably damaging/1.000 | Pathogenic | Not available |
|  | c.2009T>C(p.V670A) | TOLERANT /0.05 | probably damaging/0.873 | Unknown significance | 0.0002 |
